# LncRNA PCGEM1 in Human Cancers: Functions, Mechanisms and Promising Clinical Utility

**DOI:** 10.3389/fonc.2022.847745

**Published:** 2022-02-21

**Authors:** Yuanshuai Su, Xinyu Gu, Qiuxian Zheng, Lingxiao Zhu, Juan Lu, Lanjuan Li

**Affiliations:** State Key Laboratory for Diagnosis and Treatment of Infectious Diseases, National Clinical Research Center for Infectious Diseases, Collaborative Innovation Center for Diagnosis and Treatment of Infectious Diseases, The First Affiliated Hospital, College of Medicine, Zhejiang University, Hangzhou, China

**Keywords:** lncRNA, PCGEM1, cancer, mechanism, biomarker

## Abstract

As novel members of the noncoding RNA family, long noncoding RNAs (lncRNAs) have been widely reported to function as powerful regulators in gene expression processes, including chromosome remodeling, transcription interference and posttranscriptional modification. With the rapid development of metagenomic sequencing, numerous studies have indicated that the dysregulation of lncRNAs is closely associated with diverse human diseases, especially cancers. Prostate Gene Expression Marker 1 (PCGEM1), a recently identified lncRNA, has been reported to play a crucial role in the initiation and progression of multiple tumors by interacting with pivotal regulators of tumor-related signaling pathways. In this review, we will retrospectively review the recent studies of the expression of lncRNA PCGEM1 in human cancers and comprehensively describe the underlying regulatory mechanism by which PCGEM1 functions in tumors. More importantly, based on the relationship between PCGEM1 and cancers, the potential application of PCGEM1 in clinical diagnosis, prognosis and therapeutic treatment will also be highlighted.

## 1 Introduction

Cancer is a complex human disease with multiple risk factors that involves biological processes such as genetic mutations, chromosomal remodeling and epigenetic alterations (1). To a certain extent, early diagnosis and timely treatment are the greatest challenges in the field of oncology. As research on the roles of genomic alternations and the immune system further develops, many new biomarkers or therapeutic strategies targeted to specific molecular changes or other biological characteristics have emerged such as specific molecular agonist that enables T cells to mediate tumor killing and generating immune memory more efficiently (2–4). Despite the rapid development of cancer research, the death rate of diverse malignancies remains high due to the lack of efficient interventions. Therefore, new potential molecular biomarkers and therapeutic targets with high sensitivity and specificity need to be investigated.

Long noncoding RNAs (lncRNAs), which are over 200 nucleotides in length, are a class of endogenous and non-protein-coding RNAs (5, 6). The majority of lncRNAs are expressed in particular tissues at specific times and are broadly involved in the transcriptional or posttranscriptional regulation of the expression of coding genes, including key regulators of multiple pathways (7, 8). In recent years, a variety of cancer studies have uncovered that lncRNAs can fulfil oncogenic or tumor-suppressive functions in cancer biology (9), impacting cancer cell biological characteristics such as multiplication capacity, invasiveness and motility through diverse mechanisms (10). Hence, lncRNAs have a powerful effect on the occurrence and development of human cancers. Classified from function, there are four types of lincRNAs: signaling, guide, decoy, and scaffold lncRNAs (1, 11). Signaling lncRNAs are correlated with particular signaling pathways and their expression is often accompanied by active signaling events (9). Guide lncRNAs combine with and direct regulatory protein complexes to specific loci and then regulate downstream biological events. Decoy lncRNAs bind to the target gene promoters and interact with transcription factors or suppressor (9). Scaffold lncRNAs function as a central platform for various protein complexes to connect and target to specific location and then regulate genomic activities. Intriguingly, accumulating evidence has revealed mechanism of action between lncRNAs and another type of noncoding RNA—miRNAs (12). For example, lncRNA CDC6 accelerates breast cancer progression by directly sponging miR-215, which further regulates the expression of CDC6 (13). Thoroughly investigating the features of lncRNAs will greatly expand our current knowledge of cancer biology and provide novel perspectives for oncotherapy.

As one of the earliest oncogenic lncRNAs discovered in prostate cancer (14), PCGEM1 has received increasing attention in recent years. The PCGEM1 gene is located at chromosome 2q32.3, without protein-coding capacity (14) and lncRNA PCGEM1 was found to distribute uniformly in cell nucleus and cytoplasm (15). In the past two decades, many studies have suggested crucial functions of PCGEM1 in the initiation and progression of various cancers, such as renal carcinoma and endometrial cancer (EC) (16, 17). Through diverse functional mechanisms, PCGEM1 has a large effect on downstream genes and then regulates cancer cell proliferation, invasion and apoptosis. PCGEM1 was also reported to modulate oxaliplatin resistance in hepatocellular carcinoma (HCC) (18). PCGEM1 can influence other diseases, such as osteoarthritis and asthma (19–21). More importantly, preclinical experiments and *in vitro* studies have shown the tremendous clinical potential of PCGEM1.

Herein, we summarized the recent progress regarding the dysregulation and cancer-related functions of PCGEM1 in cell lines and clinical samples of different types of cancer. Furthermore, the comprehensive specific molecular mode of action and potential clinical implications of PCGEM1 will also be discussed.

## 2 Association Between PCGEM1 and Clinicopathological Features in Cancers

In the past few years, PCGEM1 has been widely reported to be aberrantly expressed in various human cancers, such as glioma, oral carcinoma and EC. Associations between the dysregulation of PCGEM1 and clinical characteristics have also been observed in patients. In this section, we will discuss the aberrant expression of PCGEM1 in clinical samples from cancer patients with an emphasis on the correlated clinical features and cancer growth traits in tumor xenograft models (**Table 1**).

**Table 1:** Expression files of PCGEM1 and relevant clinicopathological features in various cancers.

Cancer type	Expression	Samples	Animal experiment	Clinicopathological features	Refs
PC	upregulated	/	tumor xenograft volume, tumor growth rate, tumor weight	/	(22)
PC	upregulated	60 PC tissues and adjacent normal tissues from patients	/	/	(23)
PC	upregulated	Matched PC and adjacent normal tissues from patients	/	/	(14)
PC	upregulated	/	tumor xenograft volume, tumor growth rate	/	(24)
PC	upregulated	131 primary PC tissues, 19 metastasized PC tissues and 29 normal tissues from patients	AR regulates expression of PCGEM1 in vivo	tumor stage	(15)
PC	upregulated	90 PC tissues and adjacent normal tissues from patients	/	family history of CaP	(25)
PC	upregulated	Matched PC and adjacent normal tissues from patients	tumor xenograft volume, tumor growth rate	/	(26)
PC	upregulated	Non-DRE urine from 271 PC patients	/	biopsy grade	(27)
GC	upregulated	40 GC tissues and adjacent normal tissues from patients	/	/	(28)
GC	upregulated	cancer and normal tissues from 317 GC patients and 100 healthy individuals	/	tumor differentiation, TNM stage	(29)
GC	upregulated	/	/	/	(30)
NSCLC	upregulated	NSCLC and adjacent normal tissues from 50 patients	/	/	(31)
NSCLC	upregulated	40 NSCLC tissues and adjacent normal tissues from patients	/	/	(32)
NSCLC	upregulated	NSCLC and adjacent normal tissues from 48 patients	/	lymph node metastasis, TNM stage	(33)
Cervical carcer	upregulated	/	/	/	(34)
Cervical carcer	upregulated	68 GC tissues and adjacent normal tissues from patients	/	FIGO stage, lymph node, distant metastasis and prognosis	(35)
EC	upregulated	95 EC tissues and 27 normal tissues from patients	tumor xenograft volume, tumor growth rate	tumor stage	(17)
Ovarian Carcinoma	upregulated	50 epithelial ovarian cancer tissues and 14 normal tisseus from patients	tumor xenograft volume, tumor growth rate	tumor differentiation	(36)
HCC	upregulated	/	/		(18)
Oral carcinoma	upregulated	60 GC tissues and adjacent normal tissues from patients	/	tumor differentiation, TNM stage, lymph node metastasis	(37)
Glioma	upregulated	43 glioma tissues and adjacent normal tissues from patients	tumor xenograft volume, tumor growth rate	WHO grades, prognosis, overall survival rate	(38)
Renal carcinoma	upregulated	renal carcinoma cancer and normal tissues from 47 patients	/	Prognose, TNM stage, tumor size and metastasis	(16)

### 2.1 Clinical Samples and Cell Lines

#### 2.1.1 Prostate Cancer

According to global cancer statistics, with approximately 1.4 million new cases and 375,000 related deaths around the world (39), prostate cancer (PC) was the second most common cancer and the fifth leading cause of cancer-related death in 2020. Initially, PCGEM1 was uncovered as an emerging noncoding RNA in prostate cancer and was found to be overexpressed in a significant proportion of tumor tissues (14). Consistently, in subsequent studies, the expression level of PCGEM1 was observed to be higher in PC tissue samples than in matched normal tissues from patients, and the same result was overserved in PC cell line experiments *in vitro* (15, 23, 26), especially in black patients and high-risk patients with a family history of PC (25). Moreover, the overexpression of PCGEM1 is positively correlated with PC initiation, progression and chemotherapy resistance, which indicates the potential tumor-related functions of PCGEM1. Notably, Parolia et al. (15) revealed that PCEGM1 was upregulated in primary PC in early stages but not in metastasized PC (15). And genes positively associated with PCGEM1 expression were significantly downregulated in higher grade PC patients from multiple independent studies. Thus, the clinical expression profile of PCGEM1 warrants further research in different tumor stages.

#### 2.1.2 Gastric Cancer

Gastric cancer (GC) ranks fourth in mortality and fifth in incidence globally (40), accounting for over 1 million new cases and approximately 769,000 deaths in 2020. On account of the lack of distinct clinical symptoms or credible biomarkers in the early stage and the poor prognosis, GC remains a major clinical challenge worldwide (41, 42). Reports from the last few years have indicated that aberrantly expressed lncRNA PCGEM1 may influence the occurrence and metastasis of GC. The expression level of PCGEM1 in GC tissues is higher than that in adjacent normal tissues (28, 29). Furthermore, the expression level of PCGEM1 is significantly correlated with tumor-node-metastasis (TNM) stage and tumor differentiation in GC (29). *In vitro* experiments also verified PCGEM1 overexpression in GC cell lines (30, 43).

#### 2.1.3 Non-Small-Cell Lung Cancer

With approximately 2.2 million new cases and 1.8 million deaths in 2020 globally, lung cancer (LC) is the major cause of cancer-related mortality (18.0% of the total cancer-related deaths) (44). Non-small-cell lung cancer (NSCLC) currently accounts for the majority of LC cases (more than 85%) (45), and the 5-year overall survival rate is below 15.9% (46). In recent years, accumulating studies have uncovered that PCGEM1 is abnormally expressed and functions as a powerful tumor regulator in NSCLC. The expression levels of PCGEM1 in NSCLC tissues are significantly higher than those in adjacent normal tissues. PCGEM1 expression have also been quantified in NSCLC cell lines and is notably upregulated compared to that in normal control cells (31–33). Moreover, PCGEM1 expression is closely associated with TNM stage (P=0.020) and lymph node metastasis (P=0.034) (33).

#### 2.1.4 Female Reproductive System Cancers

Cervical cancer (CC) and EC are two commonly diagnosed female cancers worldwide and accounted for approximately 342,000 cases and 97,000 deaths in 2020 (39). The *in situ* recurrence rate is more than 17% in CC patients, and the 5-year survival rate is less than 20% (47, 48). The 5-year survival rate of patients with stage IV EC is merely 5–15% (49). Recently, several studies have demonstrated that PCGEM1 expression is markedly upregulated in both CC and EC tissues versus normal tissues (17, 34, 35). Moreover, the overexpression of PCGEM1 is significantly associated with advanced International Federation of Gynecology and Obstetrics (FIGO) stage, lymph node and distant metastasis and a poor prognosis (35). Similarly, the PCGEM1 expression level in EC was positively correlated with the tumor stage (17). In ovarian cancer, another legal gynecological malignancy, PCEGM1 was also observed to be highly expressed in ovarian cancer tissues and PCGEM1 was higher in poor differentiation group than in well differentiation group (36).

#### 2.1.5 Other Tumors

Consistent with the above results, PCEGM1 is reported to be aberrantly upregulated in other tumors. In glioma, the most common primary malignant cancer of the central nervous system (50), the expression of PCGEM1 was significantly elevated in higher WHO grade and the lower overall survival rate of patients (38). Additionally, PCGEM1 overexpression is positively correlated with tumor differentiation, TNM stage and lymph node metastasis in both renal carcinoma and oral carcinoma (16, 37). Broadly speaking, these findings indicate the aberrant expression profiles of PCGEM1 in the different types of cancer and the crucial relation of PCGEM1 and clinicopathological characteristics of cancer, which indicates that PCGEM1 probably plays an important role in the initiation and progression of various cancers.

### 2.2 Tumor Xenograft Model

To reveal the roles of PCGEM1 in diverse cancers, an *in vivo* tumor xenograft model was established by researchers, and the effects of PCGEM1 on tumor growth (tumor volume, tumor weight and tumor growth rate, **Table 1**) were evaluated. An article published in *Nature* suggested that shRNA-mediated inhibition of PCGEM1 strongly suppressed tumor growth in a CWR22Rv1-induced PC xenograft mouse model, indicating a significant regulatory effect of PCGEM1 on the growth of castration-resistant prostate cancer (CRPC) (26). Ho et al. (22) found that 3,3’-diindolylmethane (DIM) could inhibit PC tumor growth by suppressing PCGEM1 expression in a xenograft mouse model (22). Furthermore, siRNA PCGEM1 had a potent diminishing effect on PC tumor volume, whereas PCGEM1 overexpression had an adverse effect (24). Further studies in other tumors reported that the tumor growth of the PCGEM1 groups was greater than that of the control groups in *in vivo* experiments of OC, EC and glioma (17, 36, 38).

## 3 Functions of PCGEM1 and Underlying Mechanisms

Apart from the association between dysregulated expression profiles and clinicopathological characteristics of PCGEM1 in multiple cancers, related biological effects and diverse underlying mechanisms were also explored through *in vitro* and *in vivo* experiments. Generally, PCGEM1 facilitates oncogenic pathophysiologic processes such as cancer cell proliferation and invasion through multiple axes or key modulators. In the next section, we will review the biological roles of PCGEM1 in tumors and the underlying mechanisms of PCGEM1 functions, highlighting the upstream regulators and downstream effectors in the network model. Additionally, the comprehensive functions of PCGEM1 and pivotal molecules in various tumors are listed in **Table 2**.

**Table 2:** Functions and upstream/downstream regulators of PCGEM1 in various cancer cell lines.

Cancer type	Cell lines	Upstream regulators	Target	Downstream molecules/pathways	Function	Biological effect	Refs
Renal carcinoma	HK-2, OSRC-2, ACHN, A498, 786O	/	miR-433-3p	FGF2	oncogenic	cell proliferation, migration, apoptosis	(16)
PC	LNCaP	/	/	/	oncogenic	drug susceptibility, autophagy	(51)
PC	LNCaP, LNCap95, CWR22Rv1	DIM/p54/nrb	/	AR3	oncogenic	cell apoptosis	(22)
PC	LNCaP, DU145, PC-3, PrEC	MEF2	miR-148a	/	oncogenic	cell proliferation, apoptosis	(23)
PC	RWPE-1, HEK293T, LNCaP	/	miR-145	/	oncogenic	cell proliferation, invasion and migration, apoptosis	(24)
PC	/	androgen in vivo	/	/	oncogenic	/	(15)
PC	PCGEM1, NIH3T3, LNCaP	/	/	Rb (Ser807/811)	oncogenic	cell cycle, cell proliferation, colony formation	(25)
PC	LNCaP, PC3, HEK293T	/	AR+c-Myc	Metabolic genes	oncogenic	cell growth, cell cycle progression/proliferation, apoptosis; carbohydrate metabolism, lipid synthesis, glutamine metabolism, and TCA cycle	(52)
PC	LNCaP	/	/	p53/p21	oncogenic	apoptosis	(53)
PC	LNPCaP, RWPE, WPE, LNCaP-cds1, LNCaP-cds2, CWR22Rv1	/	AR	AR target genes	oncogenic	/	(26)
PC	PC-3, DU145	cholesterol and phytosterols	/	/	oncogenic	cell proliferation, mitosis, apoptosis	(54)
PC	LNCaP, LNCaP-AR+, VCaP	PCA3	/	/	oncogenic	cell proliferation	(55)
PC	PC3, DU145, LNCaP	γ-oryzanol	/	/	oncogenic		(56)
GC	BGC-823, SGC-7901, GES-1	/	miR-129-5p	P4HA2	oncogenic	cell invasion and metastasis	(43)
GC	GSE-1, SGC-7901, BGC-823	hypoxia-responsive		SNAI1	oncogenic	cell invasion and metastasis; EMT	(28)
GC	/	/	/	/	oncogenic	/	(29)
GC	AGS, MKN45	/		SNAI1	oncogenic	cell invasion and migration; EMT	(30)
NSCLC	BEAS-2B, A549, NCI-H1299, NCI-H1650, PC-9	/	miR-433-3p	WTAP	oncogenic	cell proliferation, migration and invasion, apoptosis	(31)
NSCLC	A549, H1299, H460, H1975, BEAS-2B, HEK293T	/	miR-590-3p	SOX11	oncogenic	cell viability, proliferation, invasion and migration	(32)
NSCLC	SK-MES-1, A549, H460, H522, NHBE	/	miR-152-3p	/	oncogenic	cell proliferation, invasion and migration	(33)
HCC	Hep3B/OXA	/	miR-129-5p	ETV1	oncogenic	cell invasion and migration, cell viabililty, oxaliplatin resistance	(18)
Cervical carcer	HeLa, SiHa, Caski, H8	/	miR-642a-5p	LGMN	oncogenic	cell cycle, cell proliferation, invasion and migration	(34)
Cervical cancer	Ect1/E6E7, C33A, HeLa, SiHa, CaSki	/	miR-182	FBXW11/NF-κb+β-catenin/TCF	oncogenic	cell cycle, cell proliferation, invasion and migration, EMT	(35)
Ovarian Carcinoma	A2780, OVCAR3	/		RhoA/YAP, MMP2, Bcl-xL, P70S6K	oncogenic	cell proliferation, invasion and migration, cell apoptosis	(36)
Oral carcinoma	OMEC, KB, BcaCD885, SCC-4, CAL27, SCC-15	/	miR-148a	TGFβ2/Smad2	oncogenic	cell proliferation, invasion and migaration	(37)
Glioma	U251, U-87, LN-229, NHA	/	miR-539-5p	CDK6	oncogenic	cell growth, proliferation, colony formation, invasion and migration	(38)
EC	RPMI-1640, DMEM, Ishikawa, HEC-1B	/	miR-129-5p	STAT3	oncogenic	cell proliferation, invasion and migration, apoptosis	(17)

### 3.1 Cell Growth and Apoptosis

#### 3.1.1 CeRNA Activity

**Figure 1:**
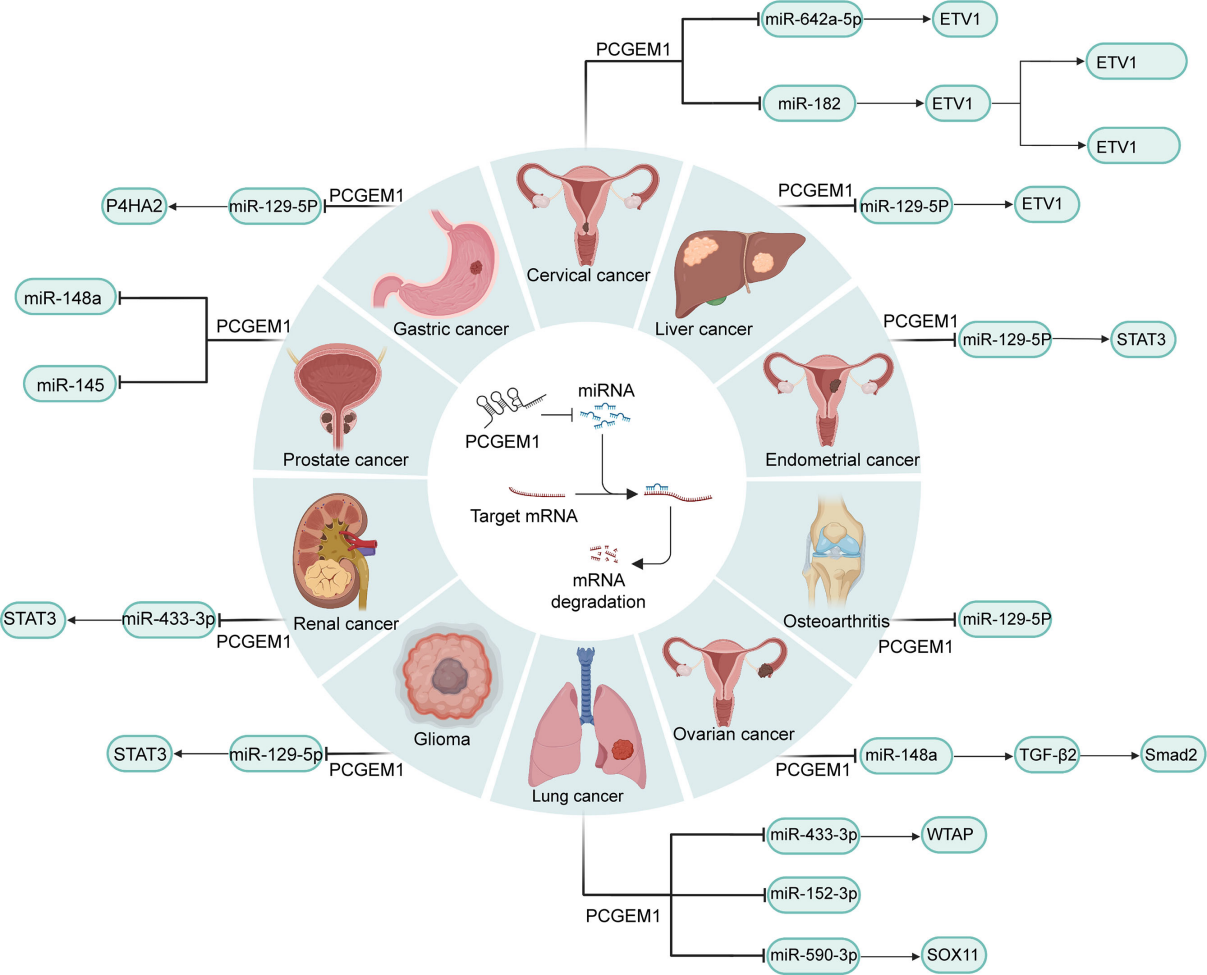
PCGEM1-miRNA-mRNA networks in various cancers. By combining with diverse miRNAs that degrade mRNAs or repress translation at a posttranscriptional level, PCGEM1 modulates the expression of key factors in tumor-related pathways, such as STAT3, Smad2 and NF-kB.

Human cancers share common characteristics descried as hallmarks, among which excessive proliferation and hypoactive apoptosis are the most prominent (57). With the progress regarding molecular biology techniques such as RNA immunoprecipitation, RNA pull-down and luciferase reporter assays, the competing endogenous RNA (ceRNA) (lncRNA-miRNA-mRNA) network has been universally acknowledged to exert a crucial impact on physiological and pathological processes that PCGEM1 mediates in cancers (**Figure 1**) (58). Some lncRNAs contain sequence motifs which could interact with the complementary regions of targeted miRNAs which regulate genes by suppressing protein translation or degrading target mRNAs through binding to targeted mRNAs (59, 60). Hence, these lncRNAs compete with targeted mRNAs and release the negative regulatory effect of miRNA on mRNA. Cai et al. (16) found that PCGEM1 in renal carcinoma cell lines could interact with miR-433-3p as a ceRNA and then upregulate fibroblast growth factor 2 (FGF2), leading to enhanced cell proliferation (16). Moreover, promoted cell apoptosis with PCGEM1 silencing was observed by Caspase-3 activity assay (16). In NSCLC cell lines, PCGEM1 was capable of modulating the expression of WT1-associated protein (WTAP) and SRY-box transcription factor 11 (SOX11) by sponging miR-433-3p and miR-590-3p (32, 61), respectively, to strongly promote cell growth. Huang et al. (33) argued that miR-152-3p might be another PCGEM1 target in NSCLC. In CC cell lines, PCGEM1 was also shown to function as a promotor of cell proliferation and cell cycle progression *via* the miR-642a-5p/LGMN axis (34). In addition, PCGEM1 is capable of modulating the NF-kB and β-catenin/TCF pathways, which play a crucial role in oncogenesis and PCGEM1 regulates these two signaling pathways *via* miR-182/F-box and WD repeat domain containing 11 (FBXW11) axis (35). Zhang et al. used a dual luciferase reporter system and revealed these two pathways were enhanced by overexpressed PCGEM1. Moreover, genes regulated by NF-κB and β-catenin/TCF were significantly upregulated by PCGEM1 which was weakened by FBXW11 silencing (35). Other researchers demonstrated that PCGEM1 facilitates cell proliferation and colony formation through the miR-148a/TGFβ2/Smad2, miR-539-5p/CDK6 and miR-129-5p/STAT3 axes in OC, glioma and EC (17, 37, 38), respectively. Moreover, PCGEM1 negatively regulates the expression of miR-145 and miR-148a and then upregulates PC cell proliferation and downregulates cell apoptosis (23, 24), but the downstream target genes of this axis remain to be explored. In general, interactions with diverse miRNAs are of paramount importance in the oncogenic functions of PCGEM1 (**Figure 1**).

#### 3.1.2 Scaffolding Activity

**Figure 2:**
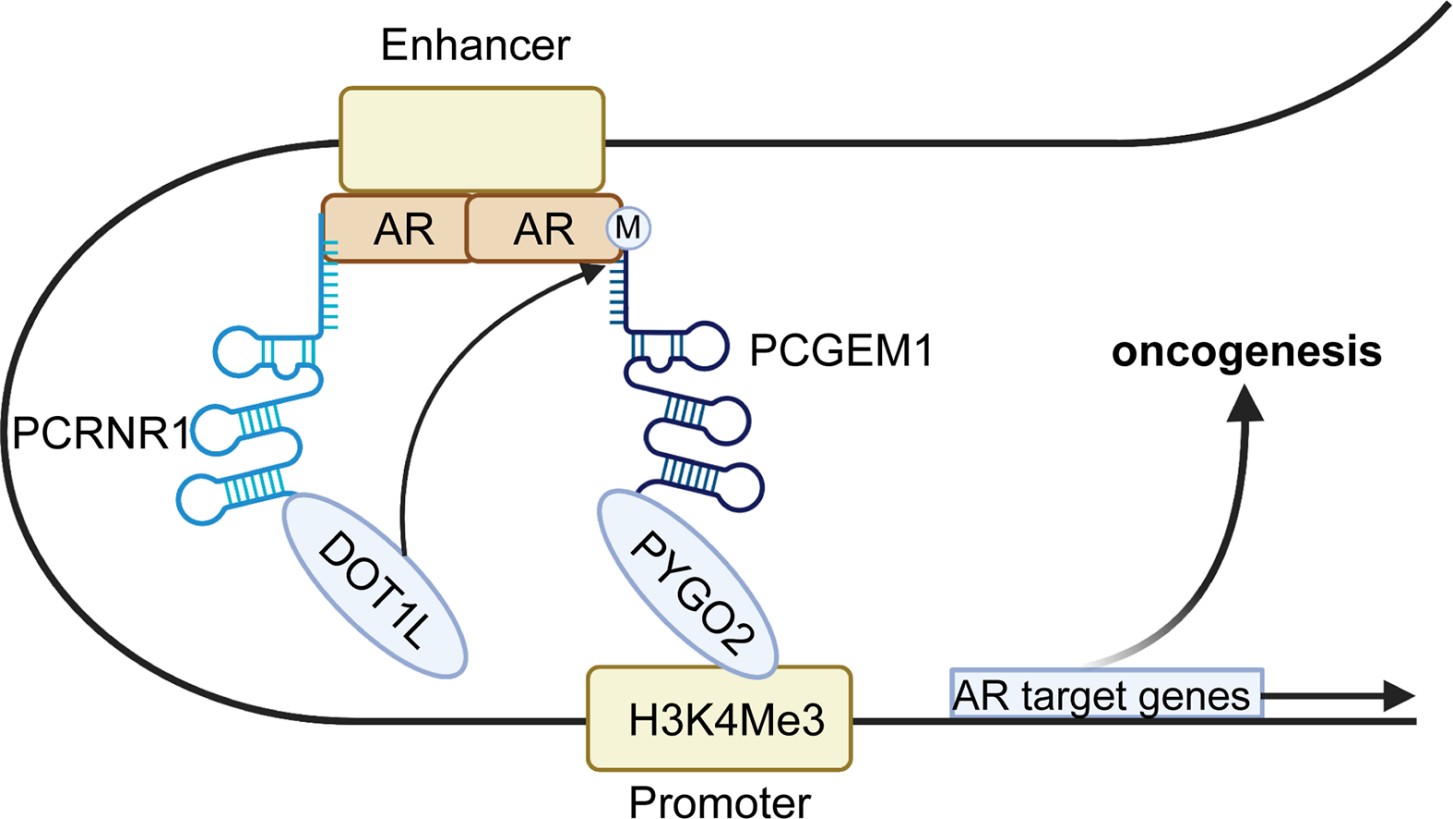
Mechanism by which PCGEM1 mediates AR target gene transcription. First, PRNCR1 combines with the acetylated AR on the enhancer and subsequently recruits DOT1-like histone H3K79 methyltransferase (DOT1L), which induces AR methylation at K349. Later, PCGEM1 is recruited to the AR and enhances PYGO2 to recognize a canonical promoter histone mark (H3K4me), thereby stabilizing enhancer-promoter looping to contribute to AR gene transcription and oncogenesis.

Endonuclear functions of PCGEM1, e.g. interacting with transcription factors, chromatin looping and hindering DNA repair are also key aspects of diverse cellular processes. Androgen receptor (AR) is an essential transcription factor for many central genes regulating prostate cell growth, and the AR signaling pathway plays a crucial role in the occurrence and development of PC (62). Hence, AR pathway inhibitors have achieved favorable results in most cases and are the long-standing first-line treatment for PC (63). However, the AR transduction pathway can function in a ligand-independent manner when PC became castration-resistant after initial androgen-deprivation treatment (26). Some studies demonstrated a close association between PCGEM1 and AR signaling. Functional assays in PC cell lines revealed the oncogenic roles of PCGEM1, indicated by the enhancement of proliferation and colony formation and the inhibition of apoptosis (22, 25, 53, 64). In terms of the mechanism (**Figure 2**), Yang et al. (26) demonstrated that PCGEM1 cooperates with another lncRNA, PRNCR1, in AR-targeted gene transcription. Further studies revealed that PRNCR1 combines with the acetylated C-terminus of AR enhancers and then recruits DOT1-like histone H3K79 methyltransferase (DOT1L), which subsequently methylates AR at K349 in the N-terminus and links PCGEM1 to AR. PCGEM1 also enhances the recruitment of pygopus family PHD finger 2 (PYGO2) to the enhancer-promoter loop, were it can interact with a typical histone promoter mark—H3 lysine 3 trimethylation (H3K4me), leading to the transcription of AR target genes (26). Nevertheless, a subsequent study indicated that PCGEM1 neither associated with CRPC nor combined with AR (65). Regardless of this, the crucial roles of PCGEM1 have been studied extensively in PC cells.

**Figure 3:**
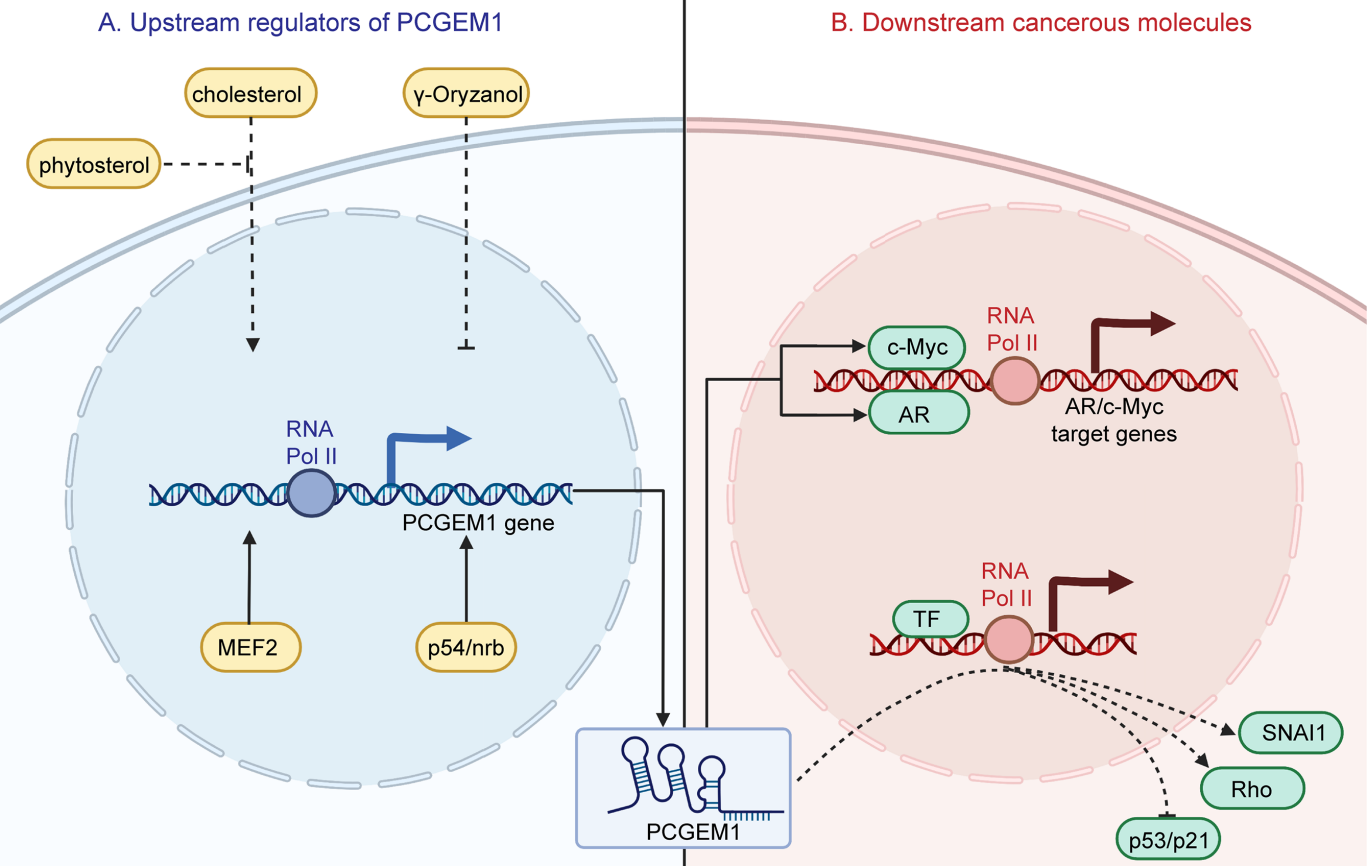
Upstream regulators of PCGEM1 and their effects on downstream cancerous molecules. **(A)** Cholesterol upregulates PCGEM1 expression, which could be reversed by phytosterol. In addition, γ-oryzanol downregulates PCGEM1. MEF2 and p54/nrb could promote PCGEM1 expression at the transcriptional level. **(B)** Regarding downstream effects, PCGEM1 could interact with AR and c-Myc to promote target gene expression. Moreover, overexpressing PCGEM1 promotes the expression of SNAI1 and Rho and delays the induction of p53/p21.

Intriguingly, PCGEM1 transcription could be upregulated by myocyte enhancer factor 2 (MEF2) and p54/nrb which enhance the activity of the PCGEM1 promoter (22, 23) (**Figure 3**). Moreover, Parolia et al. (15) observed that PCGEM1 was significantly downregulated after castration and upregulated upon AR activation *in vivo*; no such phenomenon was observed *in vitro*, indicating different transcriptional procedures *in vivo* and *in vitro* (15). Another functional study demonstrated that PCGEM1 expression could be upregulated by cholesterols even in androgen-insensitive PC cell lines; this promoted cell growth and motility, which could be reversed by phytosterols (54).

### 3.2 Cell Motility

Metastasis is the dominant cause of advanced tumor stage (66), and enhanced cell invasion and migration and PCGEM1 overexpression have been observed in diverse cancer cell lines. Zhang et al. (43) revealed that PCGEM1 facilitates GC cell invasion and metastasis *via* the miR-129-5p/prolyl 4-hydroxylase subunit alpha 2 (P4HA2) axis (43). In HCC, PCGEM1 silencing significantly suppressed the motility of Hep3B/OXA cells. Mechanistically, PCGEM1 acts as a molecular sponge of miR-129-5p to upregulate ETS variant 1 (ETV1) expression (18). Epithelial-mesenchymal transition (EMT), an essential trigger of cell invasion and migration, is closely associated with cancer progression (67, 68). Further functional assays in GC cells demonstrated that PCGEM1 promoted invasion and motility of GC cells through regulating SNAI1 (28, 30), a transcription factor that modulates the E-cadherin/N-cadherin ratio and induces EMT (69) (**Figure 3**). Piao et al. (30) found that mRNA levels of SNAI1 were not altered by the PCGEM1, but protein levels of SNAI1 were elevated as PCGEM1 was overexpressed. And then they found that the stability of SNAI1 protein significantly increased in GC cells co-cultured with exosomes that were rich in PCGEM1.

### 3.3 Metabolism

For the most part, dysregulated metabolism is interwoven with the fundamental hallmarks of cancers, either as a cause or as a consequence (70). For example, the resistance of cancer cell mitochondria to apoptosis-related permeabilization is closely associated with the variant contribution of these organelles to cancer cell metabolism (71). Cancer-cellular activities require more energy and biosynthetic activity to generate multiple macromolecular complexes throughout the cell cycle (72). Hence, it is not surprising that the metabolic activities of cancer cells and normal cells are completely disparate. Hung et al. (52) indicated that PCGEM1 regulates multiple metabolic genes and subsequently affects diverse metabolic pathways, including carbohydrate metabolism, lipid synthesis, glutamine metabolism and the tricarboxylic acid (TCA) cycle. In terms of mechanism, PCGEM1 combines with the promoters of metabolic genes and enhances the recruitment of c-Myc (73, 74), which is implicated in modulating cellular metabolism as a significant transcription factor, inducing alterations of metabolic processes at the transcriptional level (52) (**Figure 3**).

## 4 Clinical Prospects of PCGEM1

Biomarkers are defined as biological molecules existing in serum, other body fluids or human tissues that could be measured and assessed to indicate biological processes and disease features. Biomarkers are principally used for disease diagnosis and prognosis evaluation, prediction of the disease tendency, and evaluation of the response to treatment, facilitating the improvement of intervention measures for patients (75, 76). The search for effective biomarkers for PC has been ongoing for a few decades, and it has come a long way owing to advanced genomic technologies and tools (77, 78). However, the discovered specific biomarkers may be invalidated by tumor heterogeneity because these molecular mediators are closely correlated with cancer etiopathogenesis (79). Prostate-specific antigen (PSA) has been extensively used for PC screening and monitoring for a long time, but its sensitivity and specificity are inherently limited by the cancer concealment and nonsignificant increases in expression (80, 81). Considering these limitations, the thorough investigation of up/downstream molecules and the relation of biomarkers with tumor etiology is indispensable.

Above, we discussed the expression profiles and oncogenic roles of PCGEM1 in various tumors. As a novel noncoding RNA, it was confirmed to be overexpressed in PC, especially in African-American patients (14), and was found to be significantly associated with CRPC. The close relationship between PCGEM1 and clinical features, including tumor stage, metastasis and overall survival rate, has also been well demonstrated. Notably, PCGEM1 is highly expressed in noncancer prostate tissues of PC patients with a family history of PC (25). Xue and colleagues revealed that polymorphisms of PCGEM1 may make contribution to PCa risk in Chinese men (82). All of the above findings indicate its potential for early prevention, diagnosis and prognosis evaluation as a favorable biomarker. More encouragingly, the interaction between PCGEM1 and AR in PC has been brilliantly described (26), which provides new ideas for early detection and a novel therapeutic target for PC. From previous studies in multiple separate laboratories, there seems to be no consensus on the interaction of PCGEM1 and AR, although these controversial findings were preliminarily interpreted through *in vivo* and *in vitro* experiments (15, 26, 52, 65). The expression of PCGEM1 in peripheral blood was also measured in a study enrolling 144 patients with PC, and PCGEM1 expression was significantly higher in metastatic group than localized group. Moreover, the expression level in patients with poor prognosis was critically upregulated (83). Another study carried out in a multiracial population demonstrated that a 2-gene (PC3 and PCEGM1) expression panel in urine exosomes could differentiate aggressive PC from nonaggressive PC (27). Thus, the promising application of PCGEM1 needs more evaluation. Because of the important roles by which PCGEM1 facilitates PC progression in an AR-dependent or AR-independent manner, PCGEM1-targeted treatment is also an attractive area of study. For example, PCGEM1 silencing could increase the sensitivity of PC cells to baicalein and enzalutamide (51, 55), laying the groundwork for PC combination therapy. Further clinical trials are needed to design and assess the therapeutic effects of targeting PCGEM1. PCGEM1 is also regarded as a potential target in other tumors. For example, it could modulate oxaliplatin resistance *via* the miR-129-5p/ETV1 axis in HCC, indicating a promising strategy for combating HCC chemotherapy resistance (18). Currently, endoscopy and biopsy are the standard diagnostic approaches for GC, and their utility is confined to the invasiveness of the disease and limited medical resources (84). Jiang et al. (29) assessed PCGEM1 expression in GC patient serum and found that it could reflect the pathophysiological state of GC (29), demonstrating that this molecule might be a favorable indicator for GC diagnosis and prognosis.

## 5 Conclusion

As an important member of the lncRNA family located on chromosome 2q32, PCGEM1 has been confirmed to function as a tumor promotor in diverse tumors. In this review, we presented retrospective evidence of its upregulated expression based on data from multiple cancer cell lines and matched tumor/nontumor tissues. Additionally, tumor xenograft growth in a mouse model and clinical features in patients, such as tumor stage and metastasis, were found to be significantly associated with PCGEM1 dysregulation. Functional analysis also revealed that multiple biological effects, including proliferation, invasion and migration, apoptosis, drug resistance and metabolism of cancer cells, could be potently modulated by PCGEM1 overexpression. Thus, this oncogenic lncRNA plays a critical role in the initiation and progression of cancers. In terms of the underlying mechanisms, diverse modes of PCGEM1 action in various cancers with different regulatory factors and downstream signaling pathways or molecules have been investigated in various cancer types. Even in a given cancer type, such as PC, PCGEM1 functions in various ways, including the lncRNA-miRNA-mRNA network and interaction with transcription factors. Our comprehensive interpretation of the underlying molecular mechanism of PCGEM1 seems feasible, but some questions and challenges still exist. For example, what accounts for the differences in the PCGEM1 transcriptional process *in vivo* and *in vitro*? Are there other tumor-related factors co-acting with PCGEM1?

Overall, the exploration of PCGEM1 in the oncology field is undoubtedly in the initial stage. To comprehensively understand its biological roles at multiple tumor stages, we must perform additional studies. From the standpoint of etiology, clarification of the comprehensive signaling network of PCGEM1 will provide us with more challenges and opportunities to exploit novel strategies for early prevention, specific diagnosis, accurate treatment to improve the prognosis by targeting PCGEM1.

## Author Contributions

LL and JL designed and guided the study. YS, XG, and QZ wrote and edited the manuscript. LZ helped with reference collection. All authors read and approved the final manuscript.

## Funding

This work was funded by the National Key Research and Development Program of China (2021YFC2301800), and the National Nature Science Foundation of China (U20A20343).

## Conflict of Interest

The authors declare that the research was conducted in the absence of any commercial or financial relationships that could be construed as a potential conflict of interest.

## Publisher’s Note

All claims expressed in this article are solely those of the authors and do not necessarily represent those of their affiliated organizations, or those of the publisher, the editors and the reviewers. Any product that may be evaluated in this article, or claim that may be made by its manufacturer, is not guaranteed or endorsed by the publisher.

## Abbreviations

PCGEM1, prostate gene expression marker 1; lncRNAs, long noncoding RNAs; EC, endometrial cancer; HCC, hepatocellular carcinoma; PC, prostate cancer; GC, gastric cancer; TNM, tumor-node-metastasis; LC, lung cancer; NSCLC, non-small-cell lung cancer; CC, cervical cancer; FIGO, International Federation of Gynecology and Obstetrics; CRPC, castration-resistant prostate cancer; DIM, diindolylmethane; ceRNA, competing endogenous RNA; FGF2, fibroblast growth factor 2; WTAP, WT1-associated protein; SOX11, SRY-box transcription factor 11; FBXW11, F-box and WD repeat domain containing 11; AR, androgen receptor; DOT1L, DOT1-like histone H3K79 methyltransferase; PYGO2, pygopus family PHD finger 2; H3K4me, H3 lysine 3 trimethylation; MEF2, myocyte enhancer factor 2; P4HA2, prolyl 4-hydroxylase subunit alpha 2; ETV1, ETS variant 1; EMT, epithelial-mesenchymal transition; TCA, tricarboxylic acid; PSA, prostate-specific antigen.
